# The dose response of sufentanil as an adjuvant to ropivacaine in cesarean section for relief from somato-visceral pain under epidural anesthesia in parturients with scarred uterus

**DOI:** 10.1097/MD.0000000000012404

**Published:** 2018-09-21

**Authors:** Qiang Lu, Chun-shan Dong, Jun-Ma Yu, Hao Sun, Peng Sun, Xiang Ma, Chun Luo

**Affiliations:** Department of Anaesthesiology, Third affiliated hospital of Anhui Medical University.

**Keywords:** cesarean section, dose-response, epidural anesthesia, scarred uterus, sufentanil

## Abstract

Visceral pain is common during epidural anesthesia with mini dose local anesthetics in parturients during cesarean section. To reduce or avoid this complication caused by traction on the abdominal viscera, this study aimed to determine the 50% effective dose (ED_50_) and 95% effective dose (ED_95_) of epidural sufentanil as an adjuvant combination with local anesthetics for relief visceral pain in parturients with scarred uterus undergoing elective cesarean section.

One hundred parturients with scarred uterus undergoing elective cesarean section under epidural anesthesia were enrolled in this randomized, double-blinded, dose-ranging study. Parturients received 5, 10, 15, 20, and 25 μg epidural sufentanil as an adjuvant with 10 mL of 0.65% ropivacaine. Successful epidural anesthesia was defined as a sixth thoracic vertebra (T_6_) sensory level achieved within 20 minutes after epidural drugs administration and/or no visceral pain by traction on the abdominal viscera during the cesarean section. The ED_50_ and ED_95_ were calculated with a logistic regression model.

ED_50_ and ED_95_ of epidural sufentanil for successful of the pain-free from visceral pain were 10.7 μg [95% confidence interval (CI): 2.4–14.4 μg) and 28.1 μg (95% CI: 19.4–44.0 μg), respectively. The onset time to sensory block, maximum Bromage scale and duration of motor block were significant different with dose of sufentanil >20 μg (*P* < .05, compared with the other dose groups). With the dose of epidural sufentanil >20 μg could result in an increase of incidence of maternals’ adverse effects. Compared with a different dose of sufentanil, epidural administed sufentanil between 15 μg and 20 μg can maximize parturients’ satisfaction.

Our study showed that sufentanil could be used in combination with ropivacaine for relief from somato-visceral pain in patients with scarred uterus during elective cesarean section during epidural anesthesia, and that maximized parturients’ satisfaction could be achieved when the use of sufentanil with the dose between 15 μg and 20 μg for epidural anesthesia.

## 1 Introduction

Adequate analgesia during cesarean section is highly desirable for parturients while requiring less drug doses, leading to minimal adverse effects on the fetus or on the progress of surgery. However, the fact that parturients with scarred uterus may experience a longer surgery than the parturients whom the first time, and cesarean section requires traction of peritoneum and handling of intraperitoneal organs in which resulting in intraoperative visceral pain. In China, the technique of epidural anesthesia has substantially evolved that last 20 years, mainly with a mini dose opioids combined with local anesthetics regimen, and obtaining a ideal effects including prevents somato-visceral pain of surgery and decreasing in maternal adverse effects.^[1–3]^

Sufentanil, a lipophilic opioid, spinal administration in conjunction with a local anesthetics is widely used for pain relief in cesarean section because of the properties of its dose minimizing and adverse effects reduction.^[4,5]^ It should be clearly stressed that the central blocks effect of sufentanil in cesarean section and the side effects on the condition of a foetus or its recorded concentration in the umbilical blood were not in parallel.^[6,7]^ Therefore, we are reasonably sure that the ideal method of providing relief from somato-visceral pain is to use a combination of drugs that epidural administration of local anesthetics in combination with sufentanil for cesarean section in parturients with scarred uterus. However, to the best of our knowledge, few previous studies have determined the ideal dose of epidural sufentanil as an adjuvant to ropivacaine are associated with desirable pain relief and higher maternal satisfaction, and significantly lower incidence of adverse effects (including the incidence of nausea and vomiting, avoids the malaise and hypotension occurs) on maternal and fetal physiology so far.

This prospective, randomized, double-blind, controlled study was designed to investigate the effects of sufentanil by exhibiting dose-response relationships in combination with ropivacaine. For this purpose, we calculated logistic regression from a linear range of 5 different doses (5–25 μg) of epidural sufentanil as an adjuvant when co-administered with epidural 0.65% ropivacaine of 10 mL, to determine the median effective dose (ED_50_) and 50% effective dose (ED_95_) of epidural sufentanil for relief from somato-visceral pain in parturients with scarred uterus undergoing elective cesarean section patients.

## 2 Methods

### 2.1 Study subjects

The protocol in this study was approved by the Ethics Committee of Third Affiliated Hospital of Anhui Medical University (Ethical Committee number HFYY 2017010), and written informed consent was obtained from each parturient before study enrollment. Parturients with scarred uterus undergoing elective cesarean section under epidural anesthesia were recruited between July and December 2017. The inclusion criteria were that partutients with scarred uterus, American Society Anesthesiologists’ physical status I to II, and body mass index (BMI) <35 kg/m^2^. Exclusion criteria included a long history of opioid analgesic use or non-steroidal anti-inflammatory drugs (NSAIDs), psychiatric disorders, chronic hypertension, cardiovascular diseases, liver or kidney dysfunction, coagulation abnormality, platelet count less than 75  × 10^9^/L, high-risk pregnancy. Individuals were subsequently excluded from the study if epidural anesthesia was unsuccessful, or who had prolonged surgery (>1.5 h) or intraoperative blood loss more than 600 mL.

### 2.2 Anesthesia management

All parturients were brought to the operating theatre without premedication. Oxygen 2 liter minute^−1^ was delivered routinely via a open facial mask. All parturients had an intravenous (i.v.) catheter inserted in a peripheral arm vein and received an infusion of Ringer's solution at the speed of 10 mL.kg^−1^.h^−1^ before the start of epidural anesthesia. Standard monitoring procedure involved 5-lead electrocardiography, oxygen saturation (SpO_2_), heart rate (HR), and noninvasive blood pressure (NIBP) continued. The epidural anesthesia technique was performed at the first lumbar vertebra (L_1_)–second lumbar vertebra (L_2_) interspace with the parturient in the left lateral decubitus position. The epidural space was identified with 16 gauge Tuohy needle using the loss of resistance to air technique, and an epidural catheter was threaded 3.5 cm cophaladly into the epidural space and secured. Parturients were immediately positioned supine with left uterine displacement, and then received a test dose of 4 mL of 1% lidocaine through the epidural catheter. The anesthetic block was manifested within 5 minutes without side effects. A computer-generated randomisation table was used to divide parturients into 1 of 5 study groups (A, B, C, D, and E) to receive epidural 5, 10, 15, 20, and 25 μg sufentanil (Sufentanil Citrate; Inc, RenFu Pharmaceutical, China) respectively mixed with 10 mL of 0.65% ropivacaine (Ropivacaine Hydrochloride; AstraZenca AB) in all cases. The mixed solution for epidural anesthesia was prepared by 1 investigator who was not otherwise involved in the study. However, neither the anesthesiologist performing the anesthetic procedure and subsequent assessment and management nor the parturient was aware of the sufentanil dose administered and group allocation.

### 2.3 Data collection

Sensory block was bilaterally tested in each dermatomal level for loss to pinprick sensation at regular 2 minutes intervals for the first 20 minutes after the epidural drug administration. A success of epidural anesthesia was defined as bilateral T_6_ sensory block level to pinprick achieved within first 20 minutes. Maximum Bromage scale and the duration of the motor block were also studied in each group. Motor block in the lower limbs was graded according to the modified Bromage scale (0: able to flex extended leg at hip; 1: able to flex knee but not flex extended leg; 2: able to move foot only; 3: unable to move foot). The success of the epidural anesthesia was the primary endpoint. A failure of epidural anesthesia was defined as when a T_6_ sensory block level was not obtained within 20 minutes after epidural sufentanil mixed ropivacaine administration, and in cases of failure, supplemental anesthesia was required at the request of the parturient to complete surgery, which all cases of failure were excluded in this study. The outcome including the parturient did not experience intraoperative pain or, although they experienced a visceral referred pain or a little discomfort, epidural supplemental anesthesia was not required during surgery. After the commences of surgery, epidural anesthesia response was assessed at 5 minutes intervals until delivery, and thereafter at 10 minutes intervals until the end of the surgery. The epidural anesthesia responses including the somato-visceral block characteristics (caused by traction on the abdominal viscera), presence or absence of maternal adverse effects (including nausea and vomiting, pruritus, pyhoxaemia, hypotension, and bradycardia) were recorded during the period from the start of the surgery until the maternal sent to the ward. All parturients were asked to grade satisfaction scored from 1 to 4 (1 = not satisfied, 2 = moderately satisfied, 3 = satisfied, 4 = very satisfied) with intraoperative pain and the degree of comfort after the end of surgery,^[8]^ and the data were also recorded. Hypotension was defined a systolic blood pressure value of <90 mm Hg, or a 25% decrease in systolic blood pressure compared with the baseline values; it was treated if necessary, with i.v. boluses of ephedrine 5 to 10 mg; Bradycardia was defined as an HR value of < 60 bpm, which was treated with i.v. atropine 0.5 mg; Hypoxaemia was defined as SpO_2_ under 93%, which was treated with ventilatory support via facemask with higher oxygen flow. Neonate was determined using Apger scores at 1 and 5 min after delivery.

The primary outcome measure was the epidural anesthesia responses to the parturients for somato-visceral pain including the visceral pain caused by traction on the abdominal viscera, and parturient’ s satisfaction score; whereas, secondary outcomes was maternal adverse effects from the start of the surgery until the sent to the ward.

### 2.4 Statistical analysis

Using a MedSci Sample Size tools (MSST) test for trend in proportions, a sample size of 20 parturients in each group as obtained based on 5 groups with sufentanil dosage values of 5, 10, 15, 20, and 25 μg and proportions of success for relief from visceral pain were 0.2, 0.45, 0.75, 0.85, and 0.95, respectively.

Data were presented as mean and standard deviation (SD), or count as appropriate. Means with normally distributed were analyzed by 1-way analysis of variance (ANOVA) with *post hoc* Tukey test, medians and means with non-normally distributed were analyzed by Mann–Whitney *U* test, incidence data were analyzed by Fisher exact test. Overall satisfaction was compared among 5 groups using the Kruskal–Wallis *H* test. The ED_50_ with 95% CI of sufentanil was estimated by the probit regression as a back-up or sensitivity analysis. Statistical analysis were performed using the SPSS 13.0 for Windows statistical package (SPSS Inc., Chicago, IL). Statistical significance was defined as *P* < .05 (2-sided).

## 3 Results

One hundred parturients undergoing cesarean section were enrolled and randomly assigned into 1 of the 5 groups. All of the 100 parturients finished the study and included in the final analysis. The demographic profiles of the parturients in all the 5 groups were comparable with regard to age, weight, height, gestation age, mean duration of surgery, and 1 minute and 5 minutes neonate Apgar scores in Table 1.(*P* >.05).

**Table 1:**
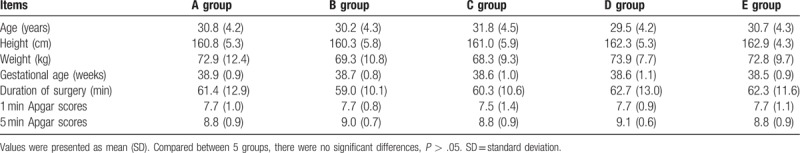
Demographic data, surgery data, and neonate Apgar scores of 5 groups (n = 20 each group).

**Figure 1:**
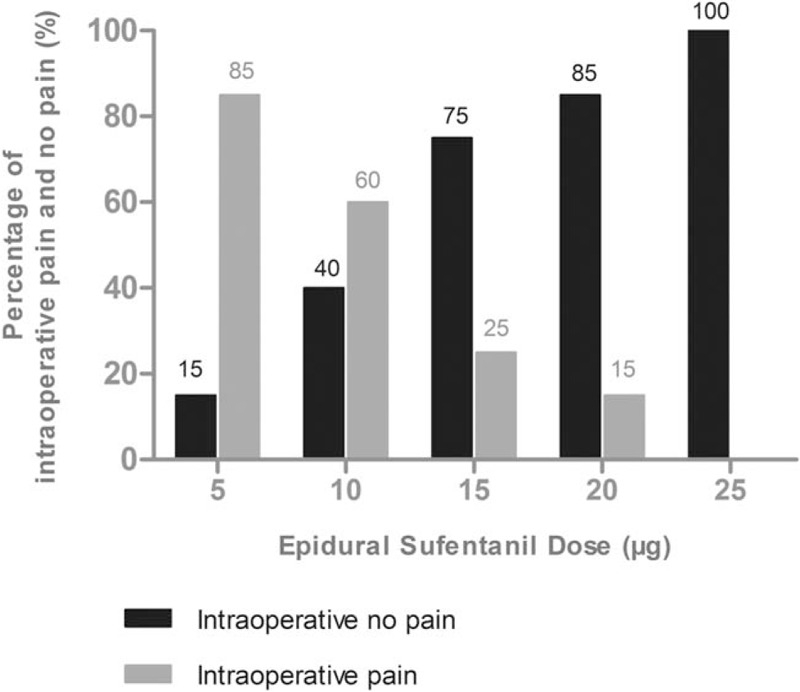
The percentages of success free pain at different doses of epidural sufentanil for the intraoperative visceral pain.

**Figure 2:**
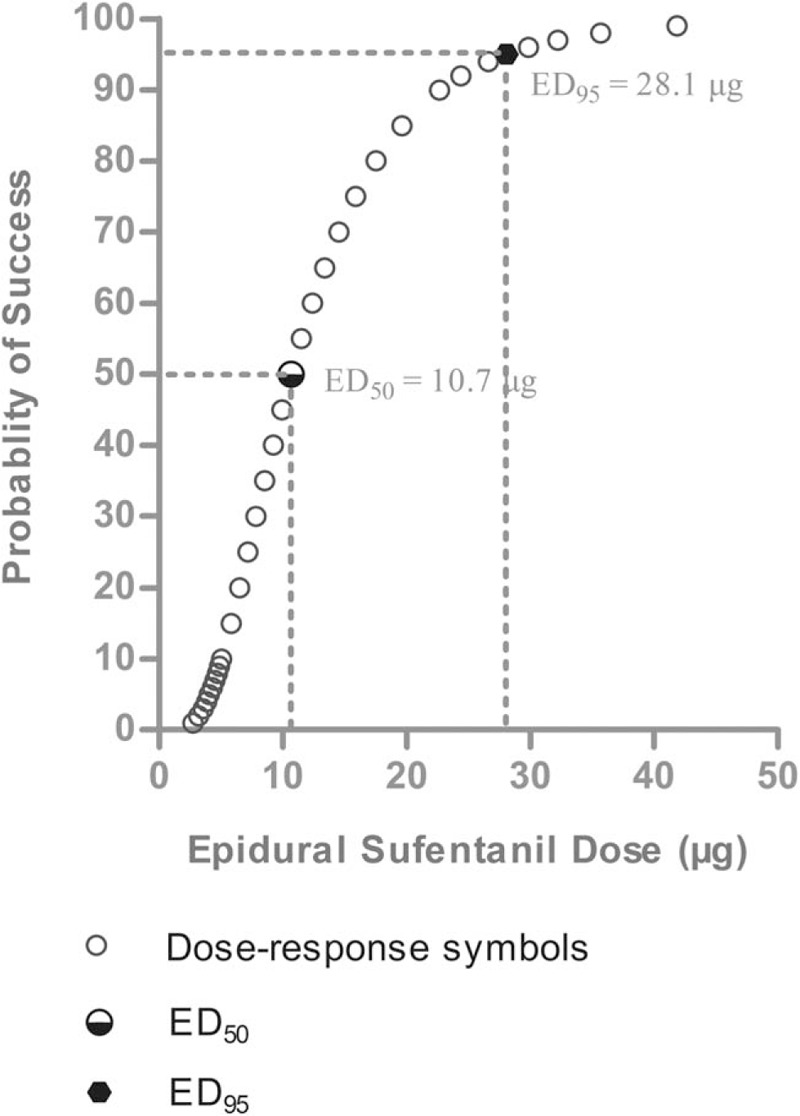
Logistic regression plot of the probability of successful epidural anesthesia versus epidural sufentanil dose. The probability of 0.5 and 0.95 was used for deriving the 50% effective dose and 95% effective dose of epidural sufentanil to achieve successful epidural anesthesia for cesarean section.

The percentages of success and failure doses of sufentanil for the intraoperative visceral pain in 5 groups are shown in Fig. 1. Logistic regression plots were drawn for the success of epidural sufentanil as in Fig. 2. The ED_50_ and ED_95_ of epidural sufentanil required for successful somatic-visceral pain controlled during cesarean section using 0.65% ropivacaine was 10.7 μg [95% confidence interval (CI): 2.4–14.4 μg) and 28.1 μg (95% CI: 19.4–44.0 μg), respectively.

The anesthetic characteristics of 5 groups are summarized in Table 2. The onset time to sensory block level is shorter in E group than in A group (95% CI: 0.077–2.323 min, *P* = .037). The time to T_6_ sensory level is shorter in D group than in A group (95% CI: 0.049–2.552 min, *P* = .042). The maximum Bromage score is higher in E group than in A group (*x*^2^ = 4.514, *P* = .034). The duration of motor block is longer in E group than in A group (U = 122, *P* = .033).

**Table 2:**
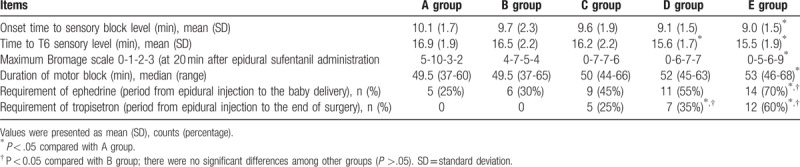
Anesthetic characteristics of 5 groups (n = 20 each group).

**Figure 3:**
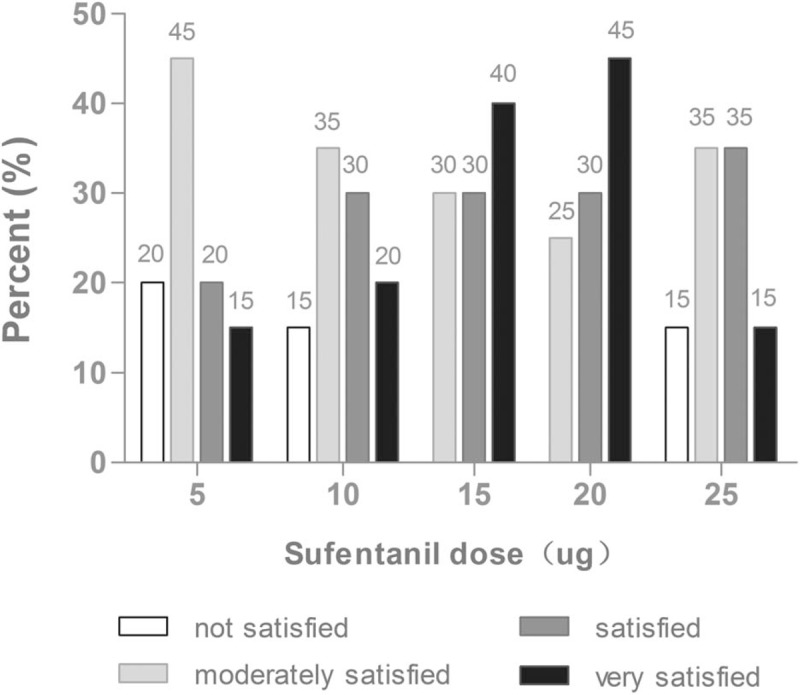
Parturients’ overall postoperative satisfaction rating at different doses of epidural sufentanil for the cesarean section. The satisfaction score was assessed using a 4-point scale (not satisfied, moderately satisfied, satisfied, and very satisfied). The 5 categories (*n* = 100, Mean Rank were 38.2, 44.0, 60.6, 66.3, and 43.6, respectively, *x*^2^ = 13.7, *df* = 4, *P* = .008).

The frequencies of maternal adverse effects at different doses of epidural sufentanil are shown in Table 3. No maternal in the A group and B group reported nausea/vomiting, fatigue, and hypotension. Nausea/vomiting were observed in 1/1 case in C group, 4/3 cases in D group, and 7/4 cases in E group, which the difference was statistically significant in the C group compared with the E group (*P* = .048). No significant differences among groups were observed regarding the frequencies of intraoperative fatigue and hypotension. No maternal in the 5 groups experienced other opioid-related adverse effects including bradycardia, hypoxaemia, and pruritus. Accordingly, the overall intraoperative satisfaction rating for parturients were different among 5 groups (*n* = 100, Mean Rank were 38.2, 44.0, 60.6, 66.3, and 43.6, respectively, *x*^2^ = 13.7, *df* = 4, *P* = .008) **(**Fig. 3).

**Table 3:**
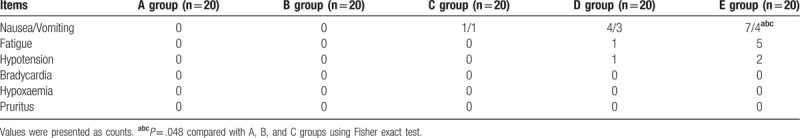
Frequency of maternal adverse effects.

## 4 Discussion

The aim of the present study was to determine the the ED_50_ and ED_95_ and clinical suitable doses for relief from somatic-visceral pain of using epidural sufentanil as an adjuvant combined with local anesthetics in cesarean section. We quantified the ED_50_ (95% CI for successful analgesia was 10.7 (2.4–14.4) μg and the ED_95_ (95% CI) was 28.1 (19.4–44.0) μg. We have demonstrated that the beneficial analgesic effects of epidural sufentanil on the ED_50_ and ED_95_ for local anesthetics in parturients with scarred uterus during cesarean section in the more doses range of 10 μg to 25 μg. However, our result also indicate that the sufentanil dose of more than 20 μg co-administered with local anesthetics for providing better introperative somatic-visceral analgesia in parturients with scarred uterus should be balanced against an increased risk of nausea/vomiting, fatigue, and hypotension or other any opioid-related adverse effects.

The addition of sufentanil to local anesthetics for intrathecal anesthesia has become a commonly used strategy for cesarean section.^[9–11]^ Sufentanil shows its expected efficacy within the dose range (2 μg-10 μg), which accelerates the action, prolongs the time of analgesia and increases the analgesic strength of block anesthetics.^[7,12]^ As sufentanil is not recommended for general anesthesia for cesarean section due to it may induce opioid-related side effects in neonates,^[13]^ such limitations do not regard central blocks as with sufentanil administered to the epidural space because of the stable anesthesia is obtained at low concentrations of sufentanil in the systemic circulation, which recommended dose is 0.5 to 1 μg.mL^−1^ for low concentrations of local anesthetics.^[7,14,15]^ Moreover, the literature supports the efficacy of epidural anesthesia with opioids versus patient-controlled epidural analgesia (PCEA) anesthesia with opioids in providing equivalent analgesia with reduced dosages of local anesthetics.^[16]^ However, for the parturients with scarred uterus, there might be adhesion in lumbosacral area of epidural space, which implies that the cerebrospinal fluid volume could be affected by the change the volume of adshesion area. A previous study has indicated that the cerebrospinal fluid volume in the limbosacral area are a most important determinants of epidural local anesthetic spread and suggests that a smaller volume cause more extensive drug spread and relatively little local anesthetics requirement for appropriately anesthesia.^[17,18]^ Because of the potential associated with sufentanil high lipid solubility, the safety of epidural administration is determined by a very large volume of distribution in the spinal cord with rapid clearance into the spinal cord vasculature,^[19]^ which implies that all the receptor effects of opioids depends on the rate and extent to which opioids distribute from the cerebrospinal fluid to the spinal cord dorsal horn and opioids are administered spinally mainly with the aim of achieving selective spinal analgesia.

There are many studies in the literature reporting the use of different doses of intrathecal ropivacaine provides reliable anesthesia in patients with scarred uterus for cesarean delivery.^[10,20]^ It is well-accepted that intrathecal administered small-dose sufentanil has a significant local anesthetic-sparing effect for cesarean section, which the most probable explanation is that a predominantly spinal mechanism by either systemic absorption or cephalad spread within the cerebrospinal fluid.^[19,21]^ This is the first study to assess the dose response of epidural sufentanil on the ED_50_ and ED_95_ for ropivacaine in parturients with scarred uterus during cesarean section. The benefits of action of sufentanil appears to be to the size of the dose by epidural administered, which means that sufentanil provides the strength of visceral sensory block with a dose-dependent manner, and the quality and duration of the pain free period are important determination for relief from both somatic and visceral pain in parturients with scarred uterus. Although the properties of the sensory block of sufentanil with ropivacaine can be explained by either the high lipid solubility or its coupled with high affinity for μ-opioid receptors,^[5]^ it is still unknown whether perineurally administered sufentanil plays a role in the course of clinical analgesic treatment by binding central or peripheral μ-1 receptors and reducing the consumption of local anesthetics, especially the analgesic efficacy and side effects rates of regional anesthesia combined with the opioids on patients during surgery or postoperative was the focus of clinical investigation over the recent years.

Cesarean section is a pelvic procedure in the lower abdomen and spinal anesthesia may completely block the sympathetic pathway, as already know, visceral pain may be manifestation from a single organ such as uterus or may be arised from algogenic conditions affecting more than 1 organ.^[22]^ From a meta-analysis may suggest that sufentanil combined with anesthetics can provide better anesthesia quality than anesthetics alone, but it is unsurprising given both the dose-dependent nature of opioid side effects and the similarities in total studies.^[23]^ Our main findings were supported by the most of similar research results to explore the study outcome of the dose response of epidural sufentanil in the intervening role. In addition, it is generally accepted that sensory analgesia to at least the fourth thoracic dermatome is necessary for cesarean section. However, even with this extent of block a substantial proportion of parturients require supplementary analgesia during exteriorization of the uterus and traction on the abdominal viscera.^[24]^ For our study, a successful block to sixth thoracic level within 20 minutes after epidural administered, and the incidence rate of relief visceral pain and opioid-related adverse effects were significantly different among groups, indicating that the cephalad spread of spinal sufentanil within epidural space would be largely restricted in the dose-dependent manner, while this results is highly suggestive for a predominantly epidural mechanism of action for a small dose of epidural sufentanil.

The main adverse effects of epidural administered sufentanil include nausea, vomiting, and pruritus, and both the fatigue and hypotension occurred only in epidural administered of sufentanil more than 20 μg in this study. Maternal hypotension, nausea, and vomiting during spinal anesthesia during and after cesarean section remain common complication.^[25]^ But the fatigue, as a common side effect of medications, 1 major mechanism by central nervous system (CNS) depression, which result in the central and peripheral inhibition of nervous conduction and release of neurotransmitters in the posterior horns of spinal cord.^[26]^ The action of sufentanil within CNS is variable; thus some papers emphasise only complete analgesia efficacy of the drug and seldom on moderate sedative and hypnotic effects. However, although literature is suggested by animal studies that show sufentanil has a mech larger spinal volume of distribution compared with other opioids,^[19]^ with an gradual increase in epidural dose of sufentanil, such protocols were reasonable when interpreting the visceral analgesia and the rate of opioid-related side effect because they maximize dose difference for epidural space administered between the sufentanil being compared.

The present study has some limitations. First, the data used in this study have an observational nature, thus this could have led to bias in documentation. However, we have no reason to suspect that documentation of intraoperative response to visceral pain in parturients were performed different depending on the dose of sufentanil that was administered during surgery. Second, from our data, we can only conclude on associations between dose-response of epidural sufentanil and relief visceral pain. Randomized controlled trials are needed to write conclusion about a causal relationship. Third, we did not measure the concentration of sufentanil in the maternal blood and cerebrospinal fluid due to limit of detection technology. Finally, we chose 10 mL of 0.65% ropivacaine in all parturient for the present study. Whether a higher dose of epidural sufentanil could result in the lower requirement of epidural ropivacaine for cesarean section in parturients with scarred uterus needs to be further studies.

## 5 Conclusion

The present study demonstrated that sufentanil could be used in combination with ropivacaine for relief from somato-visceral pain in patients with scarred uterus during elective cesarean section during epidural anesthesia, and the maximized parturients’ satisfaction could be achieved when the use of sufentanil with a dose between 15 μg and 20 μg for epidural anesthesia. Although a further increase the dose more than 20 μg of epidural sufentanil could be maximized parturients’ analgesia, it has not shown any clinical advantages in increase maternals’ side effects included nausea, vomiting, hypotension and fatigue.

## Acknowledgments

The authors would like to acknowledge the support of the Anesthesiology Research Center, Third affiliated hospital of Anhui Medical University. Also, the cooperation of the physicians and nurses of the operating room and general ward.

## Author contributions

**Qiang Lu, MD**: has made substantial contributions to conception and design, or analysis.

**Chun-shan Dong, MD, PhD**: has been involved in drafting the manuscript or revising it critically for important intellectual content and interpretation of data.

**Jun-ma Yu, MD**: agree to be accountable for all aspects of the work in ensuring that questions related to the accuracy or integrity of any part of the work are appropriately investigated and resolved.

**Hao Sun, MD**: acquisition of data

**Peng Sun, MD:** acquisition of data

**Xiang Ma, MD:** acquisition of data

**Chun Luo, MD:** acquisition of data

**Conceptualization:** Qiang Lu.

**Data curation:** Chunshan Dong.

**Formal analysis:** Chunshan Dong.

**Funding acquisition:** Chunshan Dong.

**Investigation:** Qiang Lu, Junma Yu, Hao Sun, Peng Sun, Xiang Ma, Chun Luo.

**Methodology:** Qiang Lu, Junma Yu.

**Resources:** Hao Sun, Peng Sun, Xiang Ma.

**Software:** Chun Luo.

**Writing – original draft:** Chunshan Dong.
